# Conflict and Emerging Infectious Diseases

**DOI:** 10.3201/eid1311.061093

**Published:** 2007-11

**Authors:** Michelle Gayer, Dominique Legros, Pierre Formenty, Maire A. Connolly

**Affiliations:** * World Health Organization, Geneva, Switzerland

**Keywords:** Communicable diseases, emerging, epidemiology, conflict, surveillance, outbreaks, war, perspective

## Abstract

Detection and control of emerging infectious diseases in conflict situations are major challenges due to multiple risk factors known to enhance emergence and transmission of infectious diseases. These include inadequate surveillance and response systems, destroyed infrastructure, collapsed health systems and disruption of disease control programs, and infection control practices even more inadequate than those in resource-poor settings, as well as ongoing insecurity and poor coordination among humanitarian agencies. This article outlines factors that potentiate emergence and transmission of infectious diseases in conflict situations and highlights several priority actions for their containment and control.

## Abstract 2

Public health interventions and surveillance and response systems can contribute to disease control.

An emerging infectious disease is one that is either newly recognized in a population or involves a recognized pathogen affecting new or larger populations or geographic areas (*1*,*2*). Disease emergence is influenced by ecologic and environmental changes (e.g., agriculture, deforestation, droughts, floods), human demographics and behavior (e.g., population migration, urbanization, international trade and travel), technology and industry, microbial adaptation, and breakdown in public health measures (*1*,*2*).

Conflict situations are characterized by war or civil strife in a country or area within a country. Affected populations may experience defined periods of violence (weeks to months), ongoing or recurrent insecurity in a protracted conflict (years to decades), or long-term consequences of a previous (usually prolonged) war.

Conflict may lead to the displacement of large populations into temporary settlements or camps with overcrowding and rudimentary shelters, inadequate safe water and sanitation, and increased exposure to disease vectors during the acute phase of the emergency. In protracted and postconflict situations, populations may have high rates of illness and mortality due to breakdown of health systems, flight of trained staff, failure of existing disease control programs, and destroyed infrastructure. These populations may be more vulnerable to infection and disease because of high levels of undernutrition or malnutrition, low vaccine coverage, or long-term stress. Long-term consequences of civil war can affect entire countries (such as Angola, the Democratic Republic of the Congo [DRC], or Afghanistan) because of chronic lack of investment in health, education, and public works. These conditions, which are encountered during or after war and conflict, favor emergence of infectious diseases. Examples of emerging infectious diseases in conflict situations, where several overlapping risk factors are often involved, are numerous (Figure).

**Figure:**
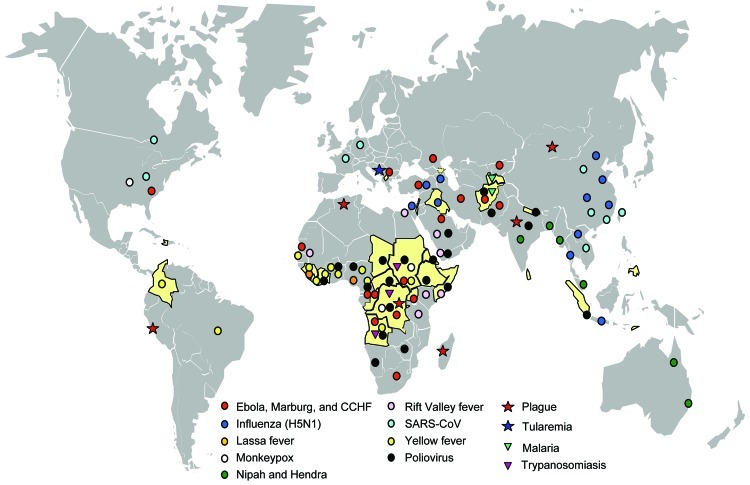
Geographic distribution of recent emerging or reemerging infectious disease outbreaks and countries affected by conflict, 1990–2006. Countries in yellow were affected by conflict during this period (source: Office for the Coordination of Humanitarian Affairs, World Health Organization, www.reliefweb.int/ocha_ol/onlinehp.html). Symbols indicate outbreaks of emerging or reemerging infectious diseases during this period (source: Epidemic and Pandemic Alert and Response, World Health Organization, www.who.int/csr/en). Circles indicate diseases of viral origin, stars indicate diseases of bacterial origin, and triangles indicate diseases of parasitic origin. CCHF, Crimean-Congo hemorrhagic fever; SARS-CoV, severe acute respiratory syndrome coronavirus.

## Risk Factors Enhancing Disease Emergence and Transmission in Conflict Situations

### Population Displacement and Environmental Conditions

Malaria had been virtually eliminated in Tajikistan in the early 1960s, and before 1992 only 200–300 malaria cases were reported annually (*3*). Civil strife during 1992–1993 led to massive population displacement and deterioration in living conditions. More than 100,000 persons fled to Afghanistan, reintroducing malaria parasites when they returned in 1994. An outbreak ensued, which reestablished *Plasmodium falciparum* malaria in Tajikistan for first time in 35 years (*4*). By 1997, 29,794 annual cases were reported, although estimates were 200,000–500,000 for that year (*3*). During 1998–1999, a reemphasis on malaria control activities reduced the incidence of malaria by 50% within 2 years (29,794 registered cases in 1997, 19,351 in 1998, and 13,493 in 1999) (*5*).

Lassa fever containment requires control of the rodent vector, good surveillance, and infection control in healthcare facilities. In West Africa, surveillance has been poor and the extent of Lassa fever is unknown. However, in the 1980s an estimated >200,000 cases and 3,000–5,000 deaths occurred annually across this region (*6*). In disease-endemic areas of Sierra Leone and Liberia, Lassa fever causes an estimated 10%–16% of hospitalizations (*7*). Civil war in the Mano River Union countries (Guinea, Liberia, and Sierra Leone) in the 1990s led to >2 million displaced persons and is likely to have provided new opportunities for rodents to proliferate when persons were forced to abandon villages and relocate in overcrowded camps. However, numbers of new cases related to the conflict are unavailable. Emergence of Lassa fever in camps in non–disease-endemic areas has been documented (World Health Organization [WHO], unpub. data) and is probably related to the poor condition of dwellings and storage of grain rations in nonsecure canvas sacks, which attracts rodents.

Similarly, unsanitary environmental conditions led to the proliferation of rats in postwar Kosovo and resulted in a tularemia outbreak among the displaced population from August 1999 through April 2000, with 327 serologically confirmed cases in 21 of 29 municipalities (*8*). The population had fled their villages because of bombings, and on their return several weeks later, they found destroyed buildings, contaminated food stores and wells, and a greatly increased rodent population. Control measures included appropriate case management, improving water and waste management, health education on hygiene, and protection of food and water sources from rats.

### Breakdown in Infection Control

Poor infection control practices in healthcare facilities have enabled amplification of outbreaks of viral hemorrhagic fevers (*9*). Medical settings have been the foci for several outbreaks of Ebola hemorrhagic fever (EHF) in Yambuku, DRC, in 1976, in Sudan in 1976 and 1979, in Kikwit, DRC, in 1995, and in Gulu, Uganda, in 2000 (*9*). Compared with other resource-poor settings, conflict situations, because of disrupted health services, may have even more substandard infection control, insufficient trained staff, and personal protective equipment (PPE), which make EHF containment difficult. The natural reservoir for this disease is present in countries affected by prolonged civil strife, and 11 of the 17 EHF outbreaks from 1976 through 2006 occurred in conflict-affected countries (*10*). Two of the largest outbreaks of EHF have been in conflict-affected countries, with nosocomial transmission playing a major role. The EHF outbreak in Kikwit, DRC, was the second largest to date with 315 cases and had a case-fatality rate (CFR) of 81% (*10*). Before infection control procedures were instituted in the hospital, 79 healthcare workers were infected compared with only 1 afterwards. These procedures included establishing an isolation facility; ensuring safe water, sanitation, and waste disposal; and providing PPE for staff (*11*). The Ebola outbreak in Gulu was the largest recorded to date (425 cases, CFR 53%), with nosocomial transmission being 1 of 3 mechanisms of spread (the others were attendance at burials and unsafe home care of EHF patients) (*12*).

The outbreak of Marburg hemorrhagic fever in Angola from October 2004 through July 2005 was the first outbreak in an African urban setting and the most lethal (374 cases, CFR 88%) (*9*,*13*). Thirty years of civil war had destroyed infrastructure, left roads mined, and left medical services with untrained staff and a persistent lack of supplies (*9*,*13*). Healthcare centers were primarily responsible for amplification of the outbreak through reuse of needles and syringes and use of multidose vials in healthcare centers due to poor training in safe injection practice (WHO, unpub. data).

Years of war in Sierra Leone during the 1990s weakened health systems and led to a long-term deterioration in infection control practices. As a result, a nosocomial outbreak of Lassa fever occurred in Kenema District Hospital from January through April 2004. A total of 410 cases occurred with a CFR of 30% (Ministry of Health Sierra Leone and WHO, unpub. data). The outbreak started in the pediatric ward, where nosocomial transmission likely resulted from use of contaminated multiuse vials and reuse of contaminated needles and syringes. Children discharged into the community were readmitted with suspected Lassa fever into the Lassa ward and comprised most of the pediatric cases in this outbreak (*14*). A total of 50% of the case-patients were <15 years of age and several deaths occurred among healthcare workers (*14*). The CFR was particularly high in young children (50% in those <5 years of age [132 cases] and 71% in those <1 year of age [41 cases]). The average CFR for Lassa fever is 1% and can be as high as 15% in hospitalized patients (*15*). During outbreaks, the CFR can reach 50% among hospitalized patients (*7*).

### Disruption of Disease Control Programs and Collapse of Health Systems

Malaria had virtually been eliminated in Afghanistan by the end of the 1970s after implementation of vector control programs in the 1960s and 1970s. However, with the onset of civil war in 1978, which continued almost without interruption until 1995, control programs collapsed and enabled malaria reemergence, including *P*. *falciparum* malaria; >50% of the population now live in malaria-endemic areas (*16*). The number of cases has been decreasing since the introduction of artemisinin-based combination therapy in the national malaria treatment protocol in 2003 (Table 1) (*17*).

**Table 1:** Officially reported malaria cases in Afghanistan, 2002–2005

Year	No. cases	*Plasmodium falciparum* confirmed
2002	629,839	83,783
2003	586,602	44,243
2004	261,456	9,212
2005	281,888	5,017

There was a significant recrudescence of sleeping sickness (human African trypanosomiasis) in the 1990s, predominantly in conflict-affected Angola, DRC, and Southern Sudan. In particular, the DRC has had a dramatic resurgence of this disease as a direct consequence of conflict. In 1930, >33,000 new cases were detected; by 1958, after active case finding and treatment, this incidence decreased to ≈1,000 new cases. Control measures were interrupted in the 1990s because of conflict, which resulted in >150,000 new cases from 1989 through 1998, with 26,000 cases in 1998 (*18*). Since 1998, detection and treatment have been reinforced in Africa, and new cases have decreased substantially amid larger populations being screened in the DRC (Table 2) (*19*). However, despite intensification of control measures, all major outbreaks in 2005 occurred in conflict-affected countries (Angola, DRC, and Southern Sudan) (*20*).

**Table 2:** New cases of trypanosomiasis per year, total population screened, and no. mobile teams for active case finding, Democratic Republic of the Congo

Year	New cases	Total screened	Mobile teams*
1930	>33,000	3,000,000	Unknown
1958	1,218	6,000,000	250
1992	5,825	525,464	4
1998	26,318	1,472,674	33
2003	10,900	2,700,000	40

*One mobile team is able to screen ≈40,000 people in 1 year.

### Inadequate Surveillance and Early Warning and Response Systems

Surveillance systems are often weak in conflict situations, which results in delays in detection and reporting of epidemics. Limited laboratory facilities and lack of expertise in specimen collection may delay confirmation of the causative organism. Outbreak investigation and implementation of control measures may be hampered by fighting, impeded access to populations, destroyed infrastructure, limited coverage of healthcare services, poorly trained health staff, and difficult logistics that prevent delivery of drugs.

An outbreak of Marburg hemorrhagic fever in Durba in northeastern DRC from October 1998 through September 2000 was the first large outbreak in rural areas under natural conditions (154 cases, CFR 83%). The area had been affected by civil war since 1997 and was controlled by Congolese rebels and Ugandan soldiers when the outbreak occurred. Although the outbreak was first reported to the national authorities in October 1998 by the chief medical officer for the health zone, an investigation was only launched after the medical officer died of this disease on April 23, 1999 (*21*). This Marburg fever outbreak was confirmed on May 6, and an international team arrived at the government’s request on May 8. Given that the area was difficult to access because of security problems and poor communications and transport infrastructure, the outbreak was already decreasing by the time the international team arrived. Only 8 cases were laboratory confirmed, and 68 were identified retrospectively by the team, which left after 3 weeks (*21*). Sporadic cases continued to occur until September 2000, although data were collected retrospectively by a second international team.

Before the implementation of the Early Warning and Response Network in Southern Sudan in 1999 by WHO in collaboration with local authorities and nongovernmental organizations (NGOs), it took 6 months to respond to a relapsing fever outbreak in 1998, which resulted in >400,000 cases and >2,000 deaths. In 2000, alerts of a relapsing fever outbreak were received within 1 week and responded to by a local team; the outbreak was contained within 2 weeks, resulting in only 154 cases and 8 deaths (*22*).

### Impeded Access to Populations

Ongoing conflict can hamper access to populations for timely delivery of supplies and implementation of control measures during an outbreak. Several outbreaks of pneumonic plague have been documented in Oriental Province in northeastern DRC, where war has hampered control efforts. Outbreaks occurred in a camp for mine workers in the Bas-Uele District (134 cases, CFR 43%) from December 2004 through March 2005 (*23*) and in the Ituri District (100 cases, CFR 19%) from May through June 2006 (*24*). In these outbreaks, achieving humanitarian access to relevant sites was difficult because of security problems, which delayed travel by response teams for investigation and implementation of control.

Access to populations to conduct vaccination campaigns may also be interrupted for months to years during protracted conflict due to long-term inadequacies in cold chain and logistics or ongoing insecurity. Low vaccine coverage has played the major role in reemergence of poliomyelitis in conflict-affected countries and has also pushed back global polio eradication targets. Conflict in Somalia since 1991 resulted in polio vaccination coverage for the required 3 polio doses being only 35% in 2005 (*25*). Somalia had been free of polio since 2002 when a large outbreak occurred in Mogadishu in 2005. By September 2006, 14 of the 19 regions in Somalia were affected with 215 cases (*26*). In May 2004, a patient infected with poliovirus was confirmed during the Darfur conflict, the first case in Sudan since 2001. By January 2005, a total of 105 cases had been confirmed in 17 of the 26 states in Sudan (*27*). Six rounds of national immunization campaigns vaccinated 8.1 million children <5 years of age in 2005, with the last case reported in June 2005. A total of 154 cases were reported in the 2004–2005 outbreak (*28*).

Interruption of routine immunization programs combined with forced migration of populations caused by conflict has also contributed to the resurgence of yellow fever in Africa (*29*). This resurgence began with the 1990 epidemic in Cameroon, then spread into conflict-affected West Africa, which since 1995 has been the most affected African region. Ten countries in Africa at risk from yellow fever have been affected by conflict, and multiple outbreaks have occurred in 6 of them: Angola (1988), Liberia (1995, 1996, 1997, 2000, 2001, and 2004), Sierra Leone (2003), Côte d’Ivoire (2000 and 2001), Guinea (2001 and 2005), and Sudan (2003 and 2005). The 2005 outbreak in Sudan resulted in a high CFR of 25% (*30*).

### Development of Drug Resistance

Pathogen resistance to drugs can contribute to disease emergence. Resistance may develop more rapidly in conflict situations because of inappropriate diagnoses or inappropriate drug regimens and outdated drugs. Treatment compliance may be poor because of purchase of insufficient quantities of drugs, selling or saving of them by patients, or interrupted treatment with sudden displacement or irregular access to healthcare facilities. In addition, private pharmacies, which can flourish in conflict situations because of no regulation, can compound this problem with drugs of unknown quality and acceptance of prescriptions from unqualified prescribers.

In an outbreak of *Shigella dysenteriae* type 1 infection in a Rwandan camp for Burundian refugees fleeing civil war in 1993, <50% of patients complied with their 5-day antimicrobial drug treatment. A high attack rate of 32% was observed among 20,000 people in that camp, with a CFR of 4%. *S*. *dysenteriae* type 1 isolated from 3 of 7 stool samples was resistant to nalidixic acid (*31*). Refugee populations had higher anti-tuberculosis (TB) drug resistance rates than nonrefugee populations in northeastern Kenya. Drug resistance to >1 drug was observed in 18% of newly diagnosed sputum-positive TB patients (with multidrug resistance in 3%) in refugee populations compared with 5% (and no multidrug resistance) in nonrefugee populations (*32*). A study of patients receiving short-course therapy for TB in an active war zone in Somalia during 1994–1995 showed that although treatment completion or cure was achieved in 70% of pulmonary TB patients, 14.5% of patients defaulted treatment (*33*), which is almost double the acceptable default rate limit for TB control programs in such settings (*34*).

### Movement of Refugees and Aid Workers

International spread of infectious diseases from conflict situations may occur through movement of refugees, relief workers, animals, goods, and private sector employees working in mining, oil, logging, or construction industries. A prolonged outbreak of hepatitis E virus in a camp in Darfur, Sudan, in May 2004 had >2,600 cases in 6 months, an attack rate of 3.3%, and a CFR of 1.7% (*35*). The outbreak occurred during an acute conflict in a setting with >1 million displaced persons crowded into camps with little access to safe water because of drought and inadequate sanitation. The outbreak subsequently spread into neighboring eastern Chad in June 2004 because of movement of Sudanese refugees fleeing Darfur.

Rebuilding and rehabilitation efforts in postconflict Sierra Leone have placed aid workers, United Nations peacekeeping forces, and businessmen at risk for contracting Lassa fever and enabled importation of cases to industrialized countries. Deaths from Lassa fever occurred in humanitarian workers in 2000, including United Nations peacekeepers (*36*,*37*). An imported case of Lassa fever was confirmed in Germany in July 2006, after the patient, a Sierra Leonean resident, flew from Freetown to Frankfurt through Abidjan and Brussels, 5 days after symptom onset (*36*). A businessman born in Liberia and residing in the United States died of Lassa fever in 2004 after traveling between Sierra Leone and Liberia before his illness (*38*). Aid workers and British soldiers have imported Lassa fever into the Netherlands (2000) and the United Kingdom (2000 and 2003) after postings in Lassa-endemic areas of Sierra Leone (*36*).

There is also a hypothetical possibility that aid workers returning from a containment zone of an emerging infectious disease, such as novel pandemic influenza, may introduce the virus causing this pandemic into conflict settings. This introduction may reduce the time for preparedness, which can lead to increased illness, death, and social disruption in these already vulnerable populations.

## Improving Detection and Control of Infectious Diseases in Conflict Situations

Detection and control of many emerging infectious diseases primarily require a functional healthcare system. This system involves investment in primary healthcare infrastructure, human resources, training, and provision of essential drugs, supplies, vaccines, and equipment. NGOs, United Nations agencies, and international organizations are providing crucial humanitarian assistance to many conflict-affected populations in coordination with relevant authorities.

In such settings, good hygiene and standard infection control precautions in health facilities are needed to reduce the potential for nosocomial transmission and amplification of disease. Correct guidance must be given on the rationale for infection control and use of PPE and isolation according to an assessment potential exposure and risk for infection. This guidance must be supported by ensuring a sustained supply of PPE, soap, disinfectants, sterilizing material, and single-use injection supplies so that shortages do not occur and force breaches in infection control.

It is imperative that the technical capacity of all humanitarian health partners and ministries of health regarding disease surveillance, prevention, and control in conflict-affected countries be enhanced to ensure effective implementation of infectious disease interventions. This implementation can be achieved through availability of internationally accepted standards, guidelines, and tools adapted to conflict situations, which can be supported by specific training of health planners and health facility staff, and rapid mobilization of international experts to provide technical field support as required. As in resource-poor settings, building the capacity of national staff must be an integral part of program implementation, especially in times of heightened insecurity, when staff often remain behind in areas and continue working.

Data on disease incidence and trends are essential for prioritizing risks and planning interventions and should be obtained through disease surveillance and early warning and response systems. Several of these systems have been implemented in conflict situations. These systems include those in Southern Sudan and for Kosovar refugees in Albania in 1999, in Darfur, Sudan, in 2004, and in Basrah Governorate, Iraq, in 2003, and resulted in early detection and response to outbreaks of EHF in Yambio in Southern Sudan in 2005, hepatitis E in Darfur in 2004, and cholera in Basrah in 2003.

Surveillance systems rely on close partnerships with NGOs, international organizations, and community groups and are built on resources and capacities of all organizations present. Effective surveillance systems in emergencies have involved selecting a small number of syndrome-based priority events, using standard surveillance forms, simplifying case definitions, health facilities weekly reporting of data, immediate reporting if set alert thresholds are passed, and establishing community mechanisms for identifying disease clusters.

Epidemic preparedness measures to be taken should involve training staff to use surveillance tools and manage cases of epidemic-prone diseases and equipping them with reliable means of communication. Isolation facilities and laboratories for pathogen confirmation must be identified in advance, and support must be provided to local institutions regarding training and supplying equipment and reagents. Mechanisms should be formulated for specimen transport and stockpiling of essential drugs, supplies, and outbreak investigation kits. Data should be analyzed locally and regular feedback provided (e.g., a weekly bulletin) to health partners. A rapid response mechanism for investigation alerts and implementation of control measures as outlined in outbreak preparedness plans (e.g., by an interagency outbreak control committee) are also crucial.

Revised International Health Regulations of 2005 provide a global legal framework to guide response to public health events of international concern. Conflict-affected countries represent one of the weakest links in global health security and should be prioritized by the international community in provision of technical and operational support to implement core capacities for detection and response to epidemics.

Military forces are increasingly implementing aid programs for conflict-affected populations. These programs have a crucial role and are a valuable resource. However, military aid can affect the neutrality of humanitarian aid. A consistent and transparent policy is needed for military humanitarian interventions, as well as extensive civil-military liaisons and close cooperation with other humanitarian agencies (*39*).

Given that healthcare in conflict situations is delivered by a wide range of national and international agencies, extensive collaboration between relevant health authorities and implementing partners should be encouraged. During an international response to an outbreak, coordination between partners and national authorities is usually ensured by WHO, which can also mobilize international experts from various institutions belonging to its Global Outbreak Alert and Response Network.

Detection, containment, and control of emerging infectious diseases in conflict situations are major challenges because of multiple risk factors that promote disease transmission and hinder control even more than those in many resource-poor settings. Beyond the global public health imperative to prevent the emergence and international spread of infectious diseases, there is also a moral imperative to alleviate the effects of these diseases on already vulnerable conflict-affected populations.
